# A Few Facts from Salter

**Published:** 1875-10

**Authors:** J. Edw. Line

**Affiliations:** Rochester, N. Y.


### ARTICLE IV.

### *A Few Facts from Salter.*

BY J. EDW. LINE, D. D. S., ROCHESTER, N. Y.

Read at the Seventh Annual Meeting of the District Dental Society oj the Seventh Judicial District of the State of N. Y., held at Rochester, N. Y., June 1st and 2d, 1875.

The author from whom we propose to draw a few facts, though well known in England, is known in this country only to those who follow, with tolerable closeness, the literature of dentistry—especially that of an imported character. If any one thing more than another served to bring his name before the dental profession, it was his war with the elder Tomes and others, on the nature of the contents of the dentinal tubuli. In this war he came off second best; but the discussion, so ably carried on by all the parties concerned, proved unexpectedly rich in fruit; for it grounded old facts on a basis firmer than before, and brought to light many other facts that might otherwise have remained unknown.

Early initiated into and made acquainted with surgery in its most comprehensive form, our author was well qualified to enter upon the practice of that specialized phase of it that we, rather pretentiously it must be said, claim to follow. For twenty-three years he has applied himself closely to dental surgery, and, as a partial result of such application, has given us a book that well merits the title—Dental Pathology and Surgery. When we add to the fact of long term of service, that he is a member of the Royal College of Surgeons, dental examiner to that institution, dental surgeon to Guy's Hospital, and that his microscope and notebook are always at his elbow, we have good reason to believe that what he has given us is the faithful record of careful maunal and intelligent mental labor.

We will first call attention to his remarks on *Secondary Dentine.* This term he regards as generic; its subdivisions —*Denrine of Repair*, *Dentine Excresence, and Osteo Dentine*—as specific. The generic term he considers "applicable to all the after-formations of dentine by which the pulp cavity is diminished or obliterated, subsequent to the tooth having attained a mature and adult condition."

1. *Dentine of Repair* is a division of our subject that has received more or less attention from Hunter down to writers of the present day; but all of these, some of the most recent observers included, "even with the assistance of the microscope, have not described many interesting points concerning its anatomy."

We are informed that "to understand the laws which are followed in the formation of dentine of repair, it is necessary to examine sections of teeth variously worn and variously broken. Sections should be made carefully, so as to cut the dentine parallel with the radius from the centre of the pulp cavity to the point of injury, and not obliquely or on one side. The injury and the corresponding point in the pulp cavity should be disclosed at once. A very slight magnifying power, from ten to twenty diameters, will suffice to exhibit the general arrangement."

*Dentine of Repair* follows or attends—

1st. *Abrasion of the surface.*

2d. *Encroachment of caries.*

3d. *Fractures that do not involve the cavity.*

1st. Experience proves that "teeth which are partially worn at the summit of their cusp, or cusps, and are likewise grooved at the neck, when cut vertically and so mounted are the most illustratize" of *abrasion of the surface.*

A canine tooth, the cusp of which has been removed by friction or by "attrition of mastication," will serve as an illustration. The enamel is wholly worn off; the dentine is in the same condition to the extent of—say an area that includes from ten to fifteen dentinal tubuli; "a large mass of tissue intervences between the wear and the repair, but yet the latter (in the state of a triangular mass of secondary dentine in the summit of the pulp cavity) has appeared, and in proportion to the former. Wear * * * has no relation to the structure of the dentine * * * but the consequent repair has a direct relation to its intimate anatomy. Now, of the tubes that abut upon the worn surface, the central would correspond to those most worn down; those which in the entire tooth had reached the extreme point of the cusp. and had consequently, by the removal of the top, been more shortened than any others. The contiguous tubes have been reduced next in amount, whilst those at the extremity of the worn surface have been least shortened. Upon tracing the tubes which have thus been rubbed down, from their outer extremities to the pulp cavity, it will be found that the amount of secondary dentine laid on, sc to speak, upon their inner extremities, is in proportion to the amount removed externally." It will be seen that "the central tubes abut upon the apex of the triangular mass of secondary dentine, while those at the sides of the wear, and next to the yet unbroken enamel, terminate internally at the edges, of the new repair tissue. And it should be observed that, not only does the repair tissue correspond with the amount of injury done to each tube, but that it is generally limited by the internal abutment of those tubes which are at the edge of the worn surface."

Teeth in which the cervical portion of the labial surface is deeply grooved, the result of one, two or three causes—defective development an acid condition of the oral secretions, friction of the tooth brush—are objects of almost daily attention. These causes show very clearly "the correspondence of the repair with the individual tubes injured. * * * The tortuous direction of the dentinal tubes in this situation, and their elevation out of the horizontal line, * * * present circumstances which admirably test and illustrate the manner in which the structural direction of the tissue determines the position of the repair."

2d. *The encroachment of caries* is a prolific cause of dentine deposit. Though Salter does not say so, it is reasonable to infer from his remarks on somewhat analogous points, that caries is almost—if not quite—always accompanied by dentine of repair. An area of carious surface that includes a dozen tubuli, points to a relative area of dentine of repair on that surface next to the pulp. This area of dentine of repair includes tha central ends of the same tubuli that open on the carious surface.

Take as an illustration a central incisor, with a cavity at the angle formed by the mesial and incisive borders. From the periphery of the tooth to that angle of the pulp chamber that corresponds to it, the tubuli converge; consequently, when the amount of dentine of repair is compared with that of tissue removed, the former shows to a disadvantage.

3d. As an illustration of what dentine of repair will do in cases of fracture, take the same tooth, the angle of which has been removed, not by disease, but by mechanical violence. It is these cases, as well as those in which the angles are removed by disease, that call for contour filling—an operation that we sometimes hesitate to perform because of the apparently near proximity of the pulp. We infer from our author that we have little reason to apprehend danger of this kind, especially in cases in which the fracture has existed for some considerable time. We may excavate directly toward the pulp with little or no fear, of exposure. All this, for the reason that the horn of the pulp opposite to the fracture has undergone calcification—has been the seat of the formation of dentine of repair.

From what has been said, "it will be seen that the dentine of repair has a very close relation both in amount and position to the injury that has caused it. When, however, the amount of wear, or fracture, or decay, is great, there ceases to be that exact and proportionate recompense, the new tissue forming over a large surface of the pulp cavity, and often much in excess; and it may be stated, as a general rule, that even where the injury is slight, if the repair be not in accurate proportion, the difference is on the side of excess, and corresponds to a larger surface than is indicated by the internal abutment of the injured tubes."

II. *Dentine Excrescences* are described as "little nodules of secondary dentine, occasionally found attached to the interior of the pulp-cavities of teeth which may be otherwise healthy, unassociated with injury or other disease. They also occur in the fangs of teeth which, higher up in the crown, are injured, and are the subjects of dentine of repair. They seldom give evidence of their presence; but it appears that sometimes they are associated with neuralgia.

III. *Osteo-dentine* "is a form of secondary dentine in which the tissue combines the characters both of bone and ivory. It is developed by the general conversion and intrinsic calcification of the several tissues of the pulp. It is usually vascular; it is frequently arranged in systems *around* vessels, like the Haversian systems in bones and it sometimes contains true lacunæ."

Every tissue of the pulp is liable to become calcified. It appears that "the vessels, or many of them, are the least affected; still they are early reduced in number, as those which occupy the axis of the dentine-Haversian systems are fár less numerous than those of the original pulp." Our author saos he has "frequently seem them of quite healthy structure when almost every trace of the other tissues has been lost."

He also tells us that osteo-dentine is less abundantly supplied with tubes "than any other form of dentine, and is usually very transparent; this does not altogether arise (as does the transparency of dentine of repair) from the filling up of the tubes with secondary deposit within them; many of the tubes are doubtless so filled up, as is the case with all dentine formed in states of tooth-irritation or inflammation; but they are nevertheless really less abundant.

What follows puts the peculiar features of the several forms of secondary dentine in a nutshell:

He says, osteo-dentine and "dentine excrescence are not infrequently seen in teeth that are worn and exhibit dentine of repair; they are evidently associated, and have a similar exciting cause. Dentine of repair, however, always forms upon that portion of the pulp-cavity next to the lesion, and is adherent, and in direct structural continuity with the primary dentine; whereas osteo-dentine and dentine excrescence occur almost always first towards the extremity of the fang, and the former is frequently quite detached from the remainder of the dentine. Moreover in dentine of repair, the pulp is detached, and can easily be removed, whereas in osteo-dentine it is bodily involved in the new tissue. Where both forms exist, they usually increase till they become confluent and confounded together, and the origin of the different forms of secondary dentine, and their relation to one another, is then lost."

IV. *The Replacement of Dentine by Masses of Bone* is a subject of some considerable interest, and opens up a broad field for speculation. One author claims that it is "probably from insufficient and neglected opportunity that I (he) must rest upon a single, but most remarkable specimen of abnormal structure to illustrate this subject."

The specimen was a molar. "It had three canals piercing the neck just below the edge of the enamel. * * * These canals were short and horizontal." It was found that the tissue around these canals was "yellower and clearer than normal dentine," and had "a cancellated aspect."

The microscope proved this "doubtful tissue * * * to be bone, resembling very closely ordinary cancellated bone of the skeleton. This occupied about a fourth of the crown and neck of the tooth. * * * Upon examining this structure with higher power of the microscope," he found it "to resemble skeleton bone more than crusta petrosa."

In this case "we have true vascular bone occupying a considerable portion of the crown of a tooth. The specimen is * * * unique. But it is very unlikely that the morbid condition has not occurred many times before. Bone structure would be liable to bone disease, and these occurring in teeth would, in such a situation, occasion anomalous and unexpected symptoms and results" He hints strongly at the probability "that some such condition may have existed in the few reported cases of abscess in the hard substance of teeth."

Such, gentlemen, are the few facts that we thought it worth while to present for your consideration. We might have extended the list indefinitely, or, perhaps, made a more judicious selection; but we felt quite sure that these few would furnish material enough for discussion, and, on the whole, prove of as much interest as any other facts embodied in the book.

Now that we are about to close our remarks, we cannot refrain from a few comments on the last fact detailed—that of replacement of dentine by bone.

When we read of this case we found ourself in a mass of queries. How happened bone in the pulp of a tooth? Is there an ultimate anatomical element in the tooth-pulp that has more than one special function? Can the connective tissue corpuscle or the dentinal cell produce other than connective tissue or dentine?

Whatever may have been the theory as regards the production of special tissues, it is now pretty well settled that special tissues are the work of special agents. Osseous tissues results from the labor of bone cells; connective tissue, from that of connective tissue corpuscles; dentine, from that of dentinal cells.

It is true that the microscope and chemical reagents, essential to histological research, give us neither physical nor chemical peculiarities, by means of which we can foretell what tissue this or that particular particle of germinal matter will produce; nevertheless, we are bound to assume, while our knowledge of this subject remains in its present condition, that any particular particle of germinal matter will produce only that kind of tissue in which it had its being, and of which tissue it once formed a part. Periosteum of bone, if removed and implanted under proper conditions, will produce in its new situation exactly what it did in its old one—it will produce bone and nothing else.

By a solution of continuity in either the hard or soft tissues, or both, the diseased elements of these tissues are liberated—are taken up by the vascular structures and conveyed to other and remote parts of the system, where, if they live at all, they infect the tissues of those parts with the same disease whose ravages gave them their freedom.

Now, possibly, in this case mentioned by Salter, there had been, at some time in the patient's history, a solution of continuity in the hard tissues; bone corpuscles, or the germinal element of such corpuscles, escaped and made their way into the vessels; were conveyed to the tooth-pulp, there to lodge and develop as true bone. Or, possibly while this tooth was in process of development, the soft pulp—at a very early stage of its history—and the elements of the cancellated structure in its vicinity, were both en closed. If this were the case, and pabulum supplied, the simultaneous development of both dentine and true bone in close relation was a foregone conclusion—*Penna. Journal of Dental Science.*

