# Correction

**Published:** 1875-12

**Authors:** 


*Correction.*—In the article published in October number of JOURNAL, entitled, "A few Facts from Salter," by Dr. J. Edward Line, on page 271 first full paragraph, fifth line, read: *The cases show, etc.*, for these causes show, etc,

